# A high prevalence of tylosin resistance among *Staphylococcus* aureus strains isolated from bovine mastitis 

**Published:** 2017-06-15

**Authors:** Farhad Bahraminia, Seyed Reza Emadi, Mohammad Emaneini, Nima Farzaneh, Mehrnaz Rad, Babak Khoramian

**Affiliations:** 1 *Department of Clinical Sciences, Faculty of Veterinary Medicine, Ferdowsi University of Mashhad, Mashhad, Iran;*; 2 *Department of Theriogenology, Faculty of Veterinary Medicine, University of Tehran, Tehran, Iran; *; 3 *Department of Microbiology, School of Medicine, Tehran University of Medical Sciences, Tehran, Iran; *; 4 *Department of Pathobiology, Faculty of Veterinary Medicine, Ferdowsi University of Mashhad, Mashhad, Iran.*

**Keywords:** Bovine mastitis, *erm* genes, Macrolides, *Staphylococcus aureus*, Tylosin resistance

## Abstract

The macrolides appear to have considerable effects for treatment of bovine mastitis because of excellent diffusion into the mammary gland, long half-life, low protein binding, high intracellular concentration and lipid solubility. Acquired resistance to macrolides in *Staphylococcus aureus* is primarily related to target-site modification through acquisition of an *erm* gene. In the present study the prevalence of both phenotypic and genotypic tylosin resistance in *S. aureus* isolates (n = 103) from subclinical mastitis in nine dairy farms belonging to three different province of Iran were investigated. Overall, *ermA*, *ermB* and *ermC* was found in 7.80%, 32.00%, and 20.40% of *S.aureus* isolates, respectively. A very high percent of isolates (56.90%) were resistant to tylosin. MIC_90_ and MIC_50_ values were 64 and 32 µg mL^-1^, respectively. Most of tylosin resistant isolates did not harbour any *erm* gene but *ermB* was dominant gene among 58 tylosin resistant isolates of *S. aureus*. In overall, tylosin resistance was prevalent in *S. aureus* isolates obtained from bovine mastitis in Iran.

## Introduction

Bovine mastitis is the most prevalent production disease affecting dairy farms worldwide, accounting for 38.00% of direct costs incurred by the dairy industry.^1^ *Staphylococcus aureus* is considered one of the most important pathogens in bovine mastitis. Effective control of *S. aureus *mastitis relies on antibiotic treatment of intramammary infections at drying-off and treatment of clinical and sub clinical mastitis during lactation.^2^

Beta-lactams, aminoglycosides, lincosamides, fluoroquinolones and macrolides are common antimicrobial compounds for treatment of *S. aureus *mastitis by the intramammary or systemic administration route. Given high bioavailability from the injection site, lipid solubility, long half-life, low protein binding and intracellular concentration in phagocytes, macrolides are of considerable interest for parenteral mastitis therapy.^1^ Tylosin and tilmicosin are routinely used for dry cow therapy in most of dairy farms in Iran.

Macrolide resistance in *Staphylococcus* species occurs by three primary mechanisms: Through target-site modification, through efflux of the antibiotics, and by drug inactivation. Target modification is predominant mechanism that is dependent on the *erm* genes.^3^ Because of extraordinary usage of macrolides for treatment of *S. aureus *mastitis in Iranian dairy farms for a long time, it is not surprising that resistance is prevalent among *Staphylococcus *species. In this study, we determined the prevalence of both phenotypic and genotypic tylosin resistance in *S. aureus *isolates from bovine subclinical mastitis.

## Materials and methods

**Bacterial isolates. **The present study was performed in nine dairy farms belonging to three different province of Iran including Tehran (three farms, n = 33), Khorasan Razavi (three farms, n = 40) and Alborz (three farms, n = 30) from 2014 to 2015. The California mastitis test (CMT) was done and milk samples were taken from quarters with score 1 or more in CMT and cultured. A total number of 103 bovine *S. aureus* isolates from more than 500 individual quarter milk with subclinical mastitis were collected. Collection of milk samples and microbiological procedures were performed according to the National Mastitis Council procedures.^4^ Isolation and identification of these presumptive *S. aureus* colonies were based on conventional methods, including Gram staining, colony morphology, production of coagulase, catalase, DNase and fermentation of mannitol. To confirm the identity of the isolate as *S. aureus*, the *nucA *gene was amplified by a PCR-based method using primers listed in Table 1. The confirmed *S. aureus* isolates were stored at –70 ˚C in brain heart broth plus 20% glycerol.

**DNA isolation and polymerase chain reaction (PCR) amplification. **For detecting macrolide resistance genes, the whole genomic DNA from cultured strains was prepared using DNA extraction kit (GeneAll, Seoul, South Korea). The PCR was carried out on the three related genes, including *ermA, ermB, ermC,* using specific oligonucleotide primers listed in Table 1. Amplification was performed in a final volume of 25 mL containing 10 μL of Taq DNA polymerase 2x master mix red containing; 2 mM MgCl_2_, Tris-HCl, (NH_4_)_2_S0_4_, 0.20% Tween 20, 0.40 mM dNTPs, 0.20 units per μL ampliqon Taq DNA polymerase inert red dye and stabilizer (Ampliqon, Odense, Denmark), 0.50 mg mL^-1^ of each primer and 3µL of template DNA. The PCR conditions consisted of a pre-denaturation step at 94 ˚C for 5 min, followed by 30 cycles of 45 sec at 94 ˚C, 50 sec at 50 ˚C (for *ermA* and *ermC* genes) or 54 ˚C (for *ermB* gene) or 55 ˚C (for *nucA*) and 75 sec at 72 ˚C. A final extension step was performed at 72 ˚C for 5 min. After amplification, DNA bands were analyzed by staining with ethidium bromide and electrophoresis (Bio-Rad, California, USA) on 1% agarose gel.

**Antibiotic susceptibility testing. **The broth micro-dilution method was used to determine minimum inhibitory concentrations (MIC), in accordance with the Clinical and Laboratory Standards Institute (CLSI).^5^ Mueller-Hinton broth was used as the test medium. Plates were incubated at 35 ˚C for 18 hr. *Staphylococcus aureus* ATCC29213 was used as reference strain for MIC quality controls. MIC were tested in the concentration range of 0.125 to 128.00 µg mL^-1^. Tylosin breakpoint 20 µg mL^-1^ was also considered in our test. ^6^

**Statistical analysis. **SPSS software (version 16.0; SPSS Inc., Chicago, USA), was used for statistical analysis. Differences in the prevalence of macrolides resistant genes and distribution of MIC values in different provinces were calculated using the chi-square test. A *p*-value of 0.05 was considered as statistically significant. 

**Table 1.** The oligonucleotide primers and the size of amplified product used in this study

**Gene**	**Primers (5'–3')**	**Size (bp)**	**Reference**
***nucA***	F- CTGGCATATGTATGGCAATTGTT	613	7
	R-TATTGACCTGAATCAGCGTTGTCT		
***ermA***	F-TATCTTATCGTTGAGAAGGGATT	139	8
	R-CTACACTTGGCTGATGAAA		
***ermB***	F-CTATCTGATTGTTGAAGAAGCATT	141	8
	R-GTTTACTCTTGGTTTAGGATCAAA		
***ermC***	F-AATCGTCAATTCCTGCATGT	299	9
	R-TAATCGTGGAATACGGGTTTG		

## Results

**Fig. 1:**
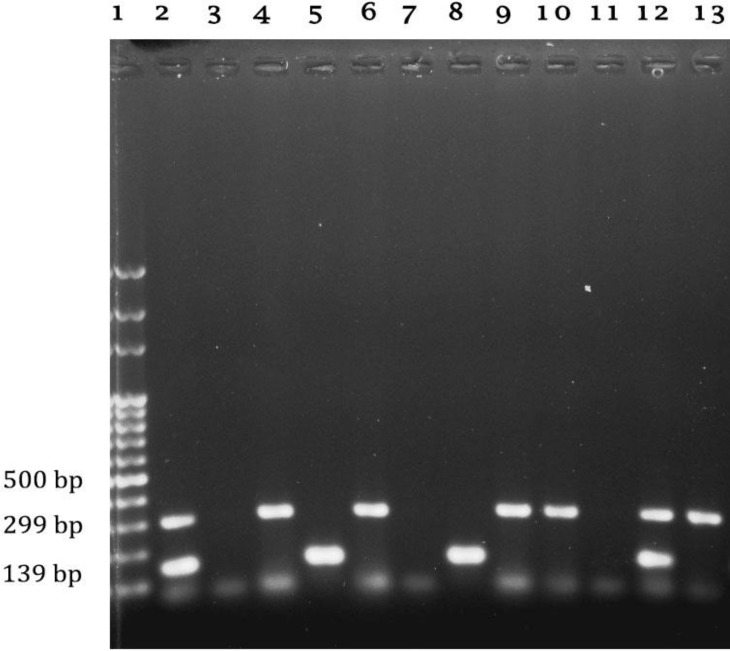
Agarose gel electrophoresis of PCR products of *ermA* and *ermC*. Lane 1: is 100 bp DNA ladder; Lane 2: Positive control, *ermA *(139 bp), *ermC *(299 bp); Lane 3: Negative control; Lane 4 to 13: samples

**Fig. 2:**
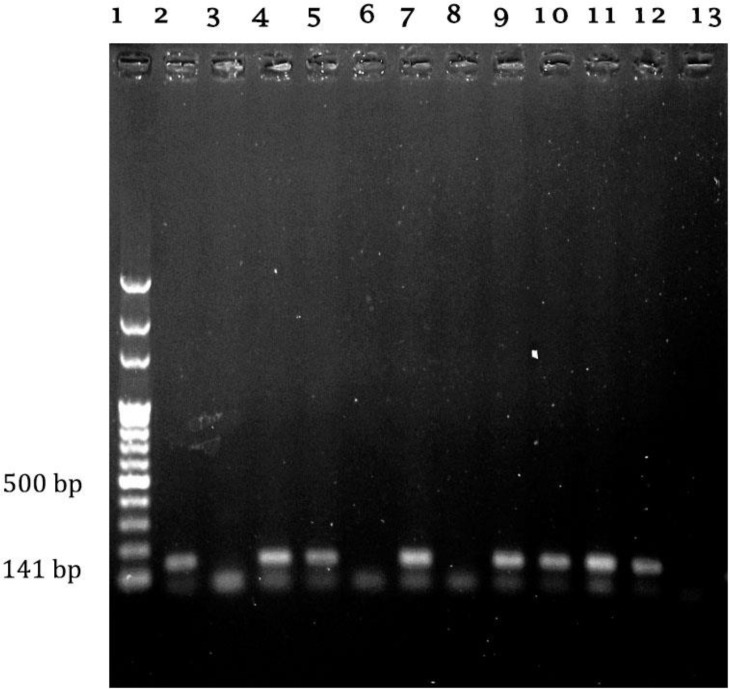
Agarose gel electrophoresis of PCR products of *ermB*. Lane 1: is 100-bp DNA ladder; Lane 2: Positive control, *ermB *(141bp; Lane 3: Negative control; Lane 4 to 13: samples

The prevalence of *S. aureus* in milk samples obtained from cows with subclinical mastitis were 22.00%, 23.50% and 25.00% in Tehran, Khorasan Razavi and Alborz, respectively. Among 103 *S. aureus* isolates 58 (56.86%) and 31 (30.09%) isolates were resistant and intermediately resistant to tylosin, respectively. The MIC values ranges for the all strains were from 0.50 to 128.00 µg mL^-1^. The MIC_90_ values in our test were 64.00 µg mL^-1^, while the MIC_50_ values were 32.00 µg mL^-1^. The distribution of MIC values in different provinces showed no significant differences (*p *> 0.05). The MIC distribution data of tylosin for the 103 *S. aureus* isolates and macrolide resistant genes are summarized in Table 2. The PCR analysis of macrolides resistant genes revealed that 8(7.80%), 33(32.00%) and 21(20.40%) isolates harboured the *ermA, ermB* and *ermC* genes, respectively (Figs. 1 and 2). 

Fifty-six isolates (54.36%) of 103 had at least one *erm* gene. The most dominant resistant gene in Tehran was *ermC* and in other provinces was *ermB*. Between provinces, *ermB* and *ermC* have significant difference (*p *< 0.05). Among 58 tylosin resistant *S. aureus*, *ermB *gene was dominant and 27.60% of isolates harboured this gene (Table 3). 

**Table 2:** Comparison of macrolides resistant genes, tylosin resistant rates (%) and MIC (µg mL^-1^) distributions of *S.* *aureus* isolates.

**Province**	**No.**	**Number (%)**			**Number of isolates inhibited by MIC **											**MIC**_50_	**MIC**_90_	**Range**	**Resistant isolates**
		***ermA***	***ermB***	***ermC***	**0.125**	**0.25**	**0.5**	**1**	**2**	**4**	**8**	**16**	**32**	**64**	**≥128**				
**Tehran**	33	1(3.03)^a^	1(3.03)^a^	7(21.21)^a^	0	0	3	0	0	1	2	5	16	5	1	32	64	0.50 - 128	66.67
**Khorasan Razavi**	40	6(15.00)^a^	21(52.50)^b^	13(32.50)^b^	0	0	0	0	1	2	6	12	9	7	3	16	64	2 - 128	47.50
**Alborz**	30	1(3.33)^a^	11 (36.67)^b^	1(3.33)^a^	0	0	4	0	1	2	2	4	9	4	4	32	≥128	0.50 - 128	56.67
**Total**	103	8(7.80)	33(32.00)	21(20.40)	0	0	7	0	2	5	10	21	34	16	8	32	64	0.50 - 128	56.86

The MIC breakpoint for tylosin (≥ 20 µg mL^-1^) was based on the veterinary antimicrobial decision support (VADS); MICs indicating susceptibility are exhibited on a white background, those indicating intermediate resistance on a light grey background, and those indicating resistance on a dark grey background.

ab Values with different superscripts in the same column are significantly different from each other (*p* < 0.05).

**Table 3:** Distribution of *erm* genes in isolates

**Genotype**	**Tylosin resistant isolates ** **(n = 58)**	**Tylosin intermediate isolates ** **(n = 31)**	**Tylosin sensitive isolates ** **(n = 14)**
***ermA*** ^+^ ***B*** ^-^ ***C*** ^-^	1 (1.70%)	0 (0.00%)	0 (0.00%)
***ermA*** ^-^ ***B*** ^+^ ***C*** ^-^	9 (15.50%)	9 (29.00%)	5 (35.70%)
***ermA*** ^-^ ***B*** ^-^ ***C*** ^+^	5 (8.60%)	3 (9.70%)	0 (0.00%)
***ermA*** ^+^ ***B*** ^+^ ***C*** ^-^	1 (1.70%)	0 (0.00%)	0 (0.00%)
***ermA*** ^+^ ***B*** ^-^ ***C*** ^+^	4 (6.90%)	0 (0.00%)	0 (0.00%)
***ermA*** ^-^ ***B*** ^+^ ***C*** ^+^	6 (10.30%)	1 (3.20%)	0 (0.00%)
***ermA*** ^+^ ***B*** ^+^ ***C*** ^+^	1 (1.70%)	1 (3.20%)	0 (0.00%)
***ermA*** ^-^ ***B*** ^-^ ***C*** ^-^	31 (53.40%)	17 (54.80%)	9 (64.30%)

## Discussion

We have determined the prevalence of macrolides resistant genes and the distribution of tylosin MICs in *S. aureus* isolates from subclinical mastitis. The prevalence of *S. aureus* isolated obtained from subclinical mastitis was 23.00%. This prevalence varies in different countries. Prevalence of S.* aureus* in China is reported to be as high as 25.20%,^10^ others reported a prevalence of 10.20% and 30.60% in Finland and Kenya, respectively.^11^^,^^12^ The same prevalence rate (25.00%) was found among nine farms in Alborz, Iran.^13^

In the present study, frequency of acquired resistance to tylosin in *S. aureus *was high (56.86%). These results were completely in agreement with other investigation who reported 56% resistance in *S. aureus *isolated from cow milk samples with clinical and subclinical mastitis in Romania.^14^ Other studies from different geographical locations reported lower resistance to tylosin ranged from 4.40% to 40.30%.^1^^,^^10^^,^^15^

Pourtaghi *et al*. determined antimicrobial resistance patterns of *S. aureus *isolated from bovine subclinical mastitis in Alborz province and showed that resistance to tylosin was 28.80% of isolates.^13^

Furthermore, the MIC_50_ value of tylosin for *S. aureus* isolates in our study was higher than those measured by previous investigators.^1^^,^^10^^,^^14^ The overall resistance of *S. aureus* isolated from bovine mastitis to macrolides in different countries is low or scarce.^16^^,^^17^ Result of present study showed higher frequency of resistance to macrolides. High resistance of *S. aureus* isolates to tylosin might be associated with the increase in highly resistant strains and rapid transfer of cloned resistance which could result from introducing and extraordinary usage of macrolides in Iranian dairy farms. When a single resistance determinant was considered, *ermB* was the most common. These observations are contrary to most other findings especially in human research that reported *ermA* was more frequent genes and *ermB* was present in only a minority of strains.^18^^,^^19^^,^^20^ Others showed that *ermC* was the most frequently encountered gene responsible for macrolide resistance among *S.aureus* isolates.^3^ Interestingly, in our investigation most of tylosin resistant isolates did not harbour any *erm* gene. It seems that other resistant genes are more important in tylosin resistance isolates obtained from bovine mastitis. Other resistant genes like *ermF*, *ermY and lunA* have also been detected in *S. aureus* isolates that were macrolide-lincosamides and streptogramin resistant.^3^^,^^21^ Also, it may suggest that one or several new resistance mechanisms for macrolides may be widespread among *S. aureus* isolates.

In conclusion, tylosin resistance was prevalent in *S. aureus* isolates obtained from bovine mastitis in Iran. The most frequent determinant of macrolides resistance was *ermB* and with *ermC* the next most common.
